# Cannabidiol binding and negative allosteric modulation at the cannabinoid type 1 receptor in the presence of delta-9-tetrahydrocannabinol: An *In Silico* study

**DOI:** 10.1371/journal.pone.0220025

**Published:** 2019-07-23

**Authors:** Hery Chung, Angélica Fierro, C. David Pessoa-Mahana

**Affiliations:** 1 Pharmacy Department, Faculty of Chemistry, Pontificia Universidad Católica de Chile, Santiago, Chile; 2 Organic Chemistry Department, Faculty of Chemistry, Pontificia Universidad Católica de Chile, Santiago, Chile; Universita degli Studi dell'Insubria, ITALY

## Abstract

Recent evidence has raised in discussion the possibility that cannabidiol can act as a negative allosteric modulator of the cannabinoid type 1 receptor. Here we have used computational methods to study the modulation exerted by cannabidiol on the effects of delta-9-tetrahydrocannabinol in the cannabinoid receptor type 1 and the possibility of direct receptor blockade. We propose a putative allosteric binding site that is located in the N-terminal region of receptor, partially overlapping the orthosteric binding site. Molecular dynamics simulations reveled a coordinated movement involving the outward rotation of helixes 1 and 2 and subsequent expansion of the orthosteric binding site upon cannabidiol binding. Finally, changes in the cytoplasmic region and high helix 8 mobility were related to impaired receptor internalization. Together, these results offer a possible explanation to how cannabidiol can directly modulate effects of delta-9-tetrahydrocannabinol on the cannabinoid receptor type 1.

## Introduction

Up to date two cannabinoid receptors are known, namely the cannabinoid receptor type 1 (CB_1_R) and the cannabinoid receptor type 2 (CB_2_R) [1,2]. Both belong to class A G-protein coupled receptors (GPCRs) and are coupled mainly to the Gα_i/0_ subunit [3]_._ The CB_1_R is abundant in the central nervous system (CNS) where it participates in the regulation of a variety of physiological and pathological conditions, including brain development, learning and memory, motor behavior, regulation of appetite, body temperature, pain perception, inflammation and it is also involved in various psychiatric, neurological, and neurodevelopmental disorders [4–6]

Agonists and antagonists of the CB_1_R have been explored as therapeutic agents for pain management and obesity, however, receptor activation has been linked to episodes of psychosis and panic while its inhibition can precipitate depressive symptoms and anxiety disorders [7]. Therefore, despite its wide therapeutic potential ligands targeting the orthosteric site of CB_1_R have failed due to CNS-related adverse effects [5,8].

Recently, three different crystal structures of the CB_1_R have been solved [9–11]. Hua *et al*, first reported the crystal structure of human CB_1_R in complex with the antagonist AM6538 (pdb ID: 5tgz) followed by the structure of the agonist AM11542-bound CB_1_R (pdb ID: 5xra). Another crystal structure of the human CB_1_R bound to the antagonist taranabant (pdb ID: 5u09) was presented by Shao *et al*. As described by Hua *et al*, important conformational changes are observed when comparing the agonist and antagonist-bound CB_1_R. Relative to the antagonist-bound state, the extracellular part of helixes 1 and 2 moves inwards by 6.6 Å and 6.8 Å respectively, while in the cytoplasmic region helix 4 moves outwards by about 8 Å. Altogether these movement lead to a 53% reduction in the volume of the ligand-binding pocket and an increase in the surface area of the G-protein-binding region. Additionally, the aromatic interaction between F200 and W356 was described as a ‘twin-toggle switch’ important for receptor activation. Aromatic stacking between F200 and W356 contributes to stabilization of the receptor in the inactive state but in the agonist-bound state, rotation of transmembrane helixes (TMH) 3 and 5 disrupts this interaction [9,10]. Together these structures provide molecular insight into the CB_1_R and can contribute to a better understanding of underlying mechanisms and interactions of the receptor with cannabinoid system orthosteric and allosteric ligands.

**Fig 1:**
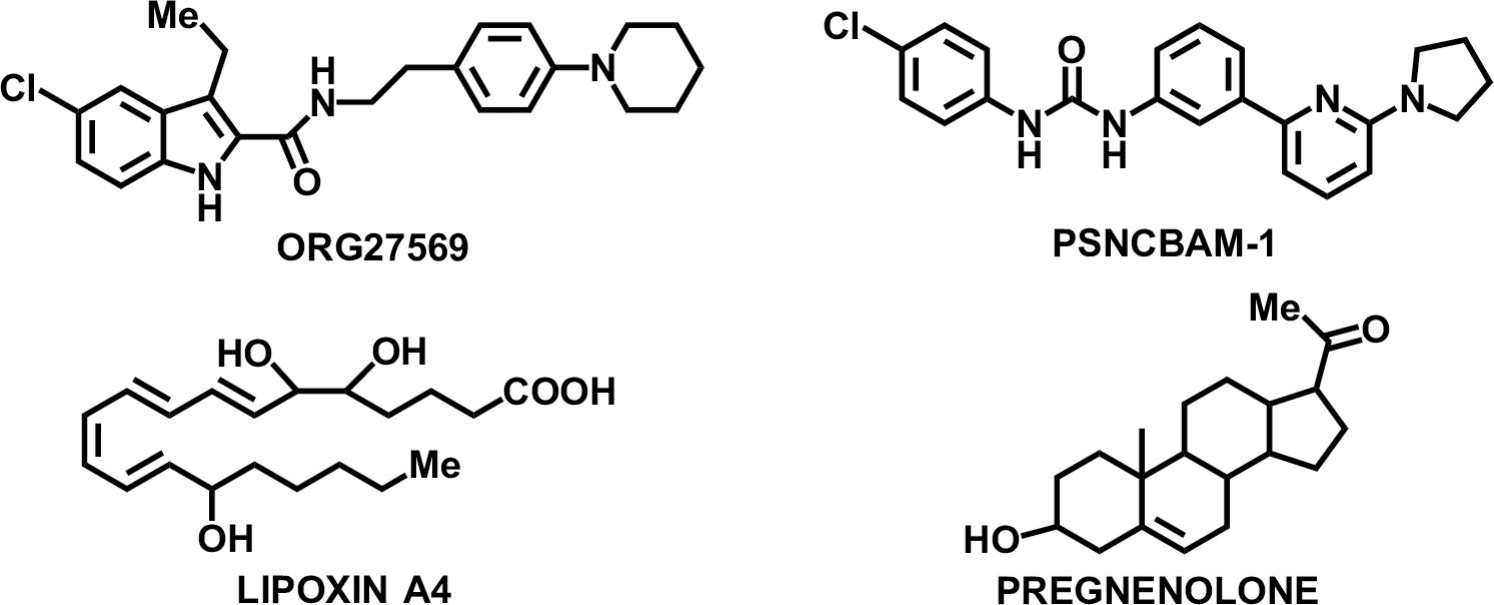
Examples of reported allosteric modulators of the CB_1_R.

Several allosteric ligands have been identified for GPCRs. The first evidence of an allosteric binding site at the CB_1_R that could recognize small molecules or allosteric modulators was reported in 2005 [12–14]. Then onwards, an increasing number of CB_1_R modulators possessing pharmacological profiles different from classical agonists and antagonists has been reported [15–22] (**Fig 1**). Allosteric sites are less conserved than orthosteric sites and show spatiotemporal specificity therefore offer the possibility of attaining safer profiles, receptor subtype selectivity and fine-tuning of receptor function [5,23]. Given the characteristic psychoactive effects of CB_1_R direct agonism, allosteric modulation represents a promising alternative that opens new therapeutic possibilities.

**Fig 2:**
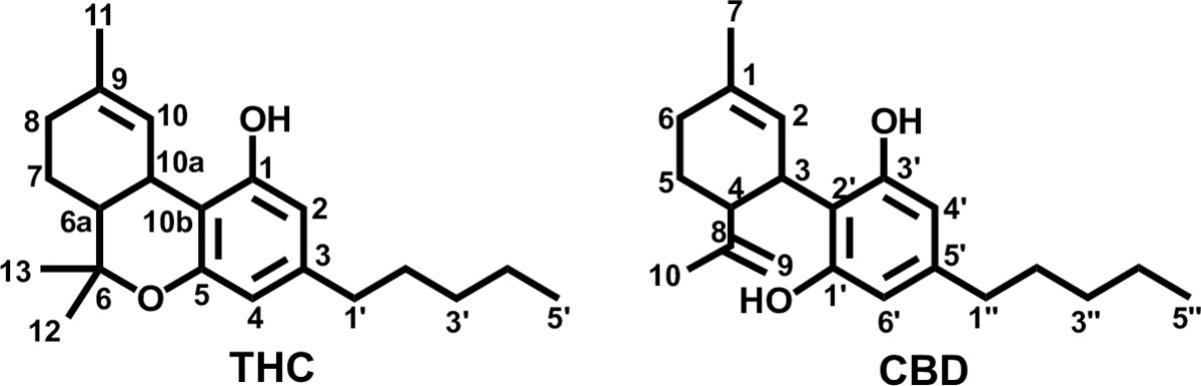
Phytocannabinoids THC and CBD.

Delta-9-tetrahydrocannabinol (THC) constitutes the main psychoactive component of the *Cannabis* plant but there is evidence that the observed therapeutic properties are not solely dependent upon the presence of THC but result from the interplay of plant cannabinoids or phytocannabinoids which participate mitigating side effects or improving the therapeutic activity [24–29]. Studies with different phytocannabinoids, particularly cannabidiol (CBD) (**Fig 2**) have reported a possible antagonic effect of this compounds over THC in the CB_1_R [30,31]. These results have raised in discussion the possibility that CBD could act as a negative allosteric modulator of the CB_1_R. Some authors suggest this phytocannabinoid can allosterically bind to the CB_1_R and thereafter modulate agonist activity [31], while others support an indirect antagonism of THC effects via mechanisms not mediated by CB_1_R [30].

The present work aims to further explore the modulation excreted by CBD upon THC effects in the CB_1_R and its possible allosteric nature. In order to do so, we have used a computational approach and the recently solved crystal structures to explore the possibility that CBD acts directly on the CB_1_R. Here we show evidence that a putative CBD binding site is located in the N-terminal region of CB_1_R, partially overlapping the orthosteric binding site. We identify receptor residues that participate in CBD binding in the presence of the agonist THC, and finally, we relate CBD binding to particular conformational changes that can impair G-protein activation. Together, these results offer a possible explanation to how CBD can directly modulate effects of THC on the CB_1_R and contribute to a better understanding of the endocannabinoid system (ECS).

## Methods

### Ligand preparation

All ligands were modelled using the Spartan’14 Software (Wavefunction, Inc. Irvine, CA). Geometry optimization calculations at the Hartree-Fock level of theory using the 6-31G* basis set was carried out using software package.

### Protein preparation

Previously reported CB_1_R crystal structures bound to agonist [10] (PDB: 5XRA) and antagonist [9] (PDB: 5TGZ) were retrieved from protein data bank. Both crystal structures were solved at 2,8 Å resolution and the CB_1_R sequence was modified to facilitate crystallization; flavodoxin was inserted as a stabilizing fusion partner within the receptor’s third intracellular loop (ICL3), the receptor was truncated on both the N and C-terminal, and four mutations (T210^3.46^A, E273^5.37^K, T283^5.47^V, and R340^6.32^E) were introduced to improve expression and thermostability. In order to prepare the protein structures, all co-crystallized heteroatoms, water molecules and flavodoxin protein were eliminated, missing residues and missing atoms were added and point mutations were converted back to the wild-type receptor sequence using UCSF Chimera package [32].

### Receptor modeling

Missing segments in the CB_1_R crystal structures were modeled using the loop modeling protocol in the Software Modeller 9v12 [33]. The N-terminal membrane proximal region (MPR*)* was modeled from residue 89 to 103 or 98, and the ICL3 from residue 306 to 336 or 331 in the crystal structures bound to agonist and antagonists respectively using the *loop*.*py* script. The disulfide bond between C98-C107 in the MPR was also modeled *model_disulfide*.*py* script. The human CB_1_R sequence was retrieved from the UniProt Knowledgebase database (UniProtKB) entry P21554. 100 runs were carried out using the standard parameters and the best model was selected based on the internal scoring function implemented in the software. The selected model was externally assessed using the free available programs ProSA [34], ANOLEA [35] and Procheck [36]

### Identification of binding cavities

Each CB_1_R generated model was mapped for potential ligand binding pockets using the web servers Computed Atlas of Surface Topography of proteins (CASTp) [37] and DoGSiteScore [38]. Out of the identified pockets only those with a volume equal or greater than CBD vdW volume (327,42 Å^​3^) were considered. One of the identified sites was selected for further molecular docking studies.

### Molecular docking

Docking studies were performed using AutoDock v4.2 [39] software suite with AutodockTools ADT 1.5.6 [39,40] following the standard docking procedure for rigid proteins. Grid maps were calculated using the autogrid option with a grid volume of 100x100x80 points with grid spacing of 0.375 Å and centered in the previously selected binding cavity. Docking simulations were performed with a Lamarckian genetic algorithm (LGA) and binding energies were estimated according to the internal scoring function implemented by the program. 250 independent runs per ligand were carried out with an initial population of 300 individuals, a maximum number of 250.000 energy evaluations, a maximum number of 27.000 generations, a mutation rate of 0.02, and a cross-over rate of 0.80. Cluster analysis of the docking conformations was conducted using a root-mean-square-deviation (RMSD) tolerance of 2.0Å. From the resulting complexes, those with the lowest free-energy binding positions were selected and further analyzed using the Visual Molecular Dynamic (VMD) visualization program [41]. Validation of the docking protocol was preformed using the co-crystallized ligands: the agonist AM11542 in the active conformation and the antagonist AM6538 in the inactive conformation of the receptor.

### System setup

Two systems were set up for MD simulations using the selected protein-ligand complexes as starting coordinates. Topology and parameter files were generated using the web server SwissParam [42]. The orientation of the receptor structures with respect to the membrane were obtained from the Orientations of Proteins and Membranes (OPM) database [43]. The protein–ligand complexes were inserted in a 1-palmitoyl-2-oleoyl-sn-glycero-3-phosphocholine (POPC) lipid bilayer with the VMD Membrane Builder plugin and solvated in a TIP3 water box with Na^+^ and Cl^-^ ions to maintain a concentration of 0.15 M. The final system size was approximately 152 Å × 153 Å × 120 Å in the active and inactive conformations.

### Molecular dynamics simulations

MD simulations were carried out in NAMD 2.9 [44] software. A 1 fs integration time step was used. Periodic boundary conditions were applied and long-range electrostatics were computed using the particle mesh Ewald algorithm [45]. For non-bonded interactions a 12 Å cutoff and a 10 Å switching distance for smoothing functions were used. Each system was energy-minimized for 10000 conjugate gradient steps. The pressure was maintained at 1 atm using the Langevin piston method and temperature was maintained at 310 K by Langevin dynamics with a damping coefficient of 5 ps^−1^. The simulations were carried out under the NPT ensemble with no fixed atoms. The total production time for the inactive and active receptor conformations was of 25 ns and 50 ns respectively. All trajectories were analyzed using the VMD visualization program [41].

## Results and discussion

Various previous studies have shown that CBD is able to modulate THC effects in the CB_1_R [30]. However, its underlying mechanism remains unclear and although a possible allosteric nature has been proposed, there is no evidence of a direct interaction between CBD and the receptor. In this work, the possibility that CBD acts directly on CB_1_R was studied using a computational approach and the recently solved crystal structures. We first modeled the missing membrane proximal region (MPR) in the N-terminal of the available CB_1_R crystal structures. A putative allosteric binding site was then identified, and molecular docking studies were carried out. Finally, molecular dynamics simulations were used to analyze specific movements that could be related to structural changes described in the receptor inactivation process.

### Molecular modeling of the CB_1_R structure

The two human CB_1_R crystal structures bound to agonist AM11542 (pdb ID: 5XRA) and to antagonist AM6538 (pdb ID: 5TGZ) were selected as they were crystalized under the same conditions and provided two different conformational states, active and inactive, in which CBD might show affinity. However, both crystallized structures lack the complete N-terminal and third intracellular loop (ICL3) regions and contain a series of stabilizing mutations. For this reason, two new models of the active and inactive receptor state were constructed based on the available crystal structures.

Previous reports have shown that most of the N-terminal tail can be deleted without affecting ligand binding. However, the highly conserved MPR–spanning residues 90 to 110– has shown to be essential for the receptors ability to bind ligand [46–48]. Furthermore, two conserved cysteine residues in the MPR (C98 and C107) form a disulfide bond that has been linked to allosteric modulation in CB_1_R. The effects of the CB_1_R allosteric ligands Org 27569 and PSNCBAM-1 was shown to be altered by the C98-C107 disulfide [46] and the allosteric activity of CBD depended upon the presence of polar residues at positions 98 and 107 [31]. Likewise, the ICL3 of the CB_1_R is known to be involved in G protein coupling and activation [49–51].

**Fig 3:**
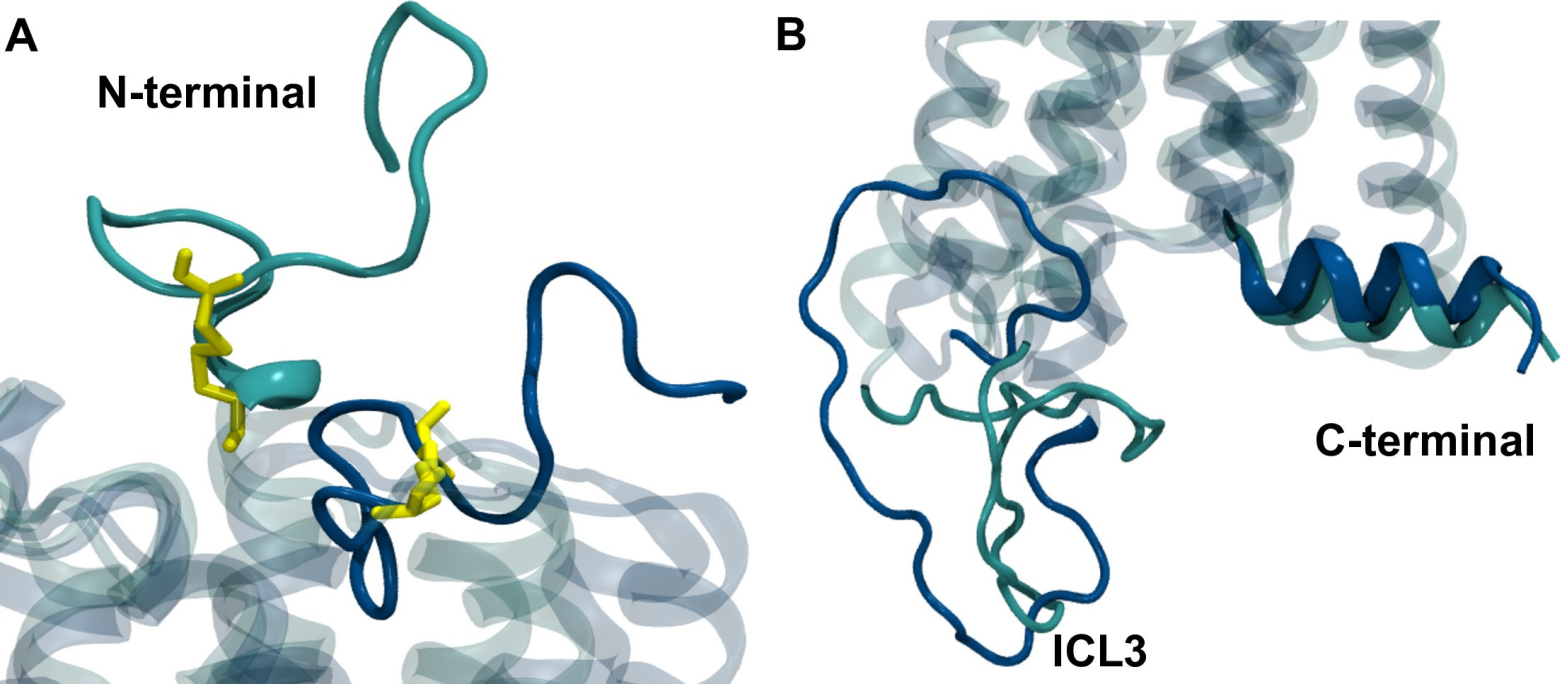
Differences observed in the CB_1_R models bound to agonist and to antagonist. (A) Difference in the N-terminal region, the C98-C107 disulfide bond is shown in yellow licorice representation. (B) Different distance between the ICL3 and the C-terminal. CB_1_R model bound to agonist is shown in cyan and to antagonist in blue.

As expected the overall 3D structures were very similar with changes primarily due to the modelled regions (**Figures A and B in S1 File**). In agreement with the crystalized structures, in the agonist-bound conformation the N-terminal region lies over the orthosteric binding site extending into the extracellular side (**Figure A in S1 File**) while in the antagonist-bound structure it creates a v-shaped turn that projects into the ligand binding site (**Figure B in S1 File**). Visual inspection reveals that in both structures the conformation of the N-terminal is constrained by the intraloop disulfide bond between C98 and C107. The v-shaped turn in the MPR is locked by this disulfide bond and seems to act as a lid above the orthosteric pocket that can open up in the active state or close back in the inactive state of the receptor and thereby modulate the access into the orthosteric site (**Fig 3A**).

Furthermore, all modelled regions (**Figures A and B in S1 File**) introduce mainly polar or charged amino acids which concentrate electronegative density in the extra and intracellular surfaces of the receptor. The rearrangement of the N-terminal residues in the inactive conformation exposes a larger electronegative surface in the membrane access channel which could in turn hinder further entry of lipidic molecules into the ligand binding site.

On the other hand, the modelled intracellular segment shows that the ICL3 coils inwards towards the C-terminal tail in the active conformation and turns away in the inactive conformation. This change is in agreement with the agonists-induced reduction in the distance between the ICL3 and the C-terminal that has been described in receptor activation [52] (**Fig 3B**).

### Identification of receptor binding sites

**Fig 4:**
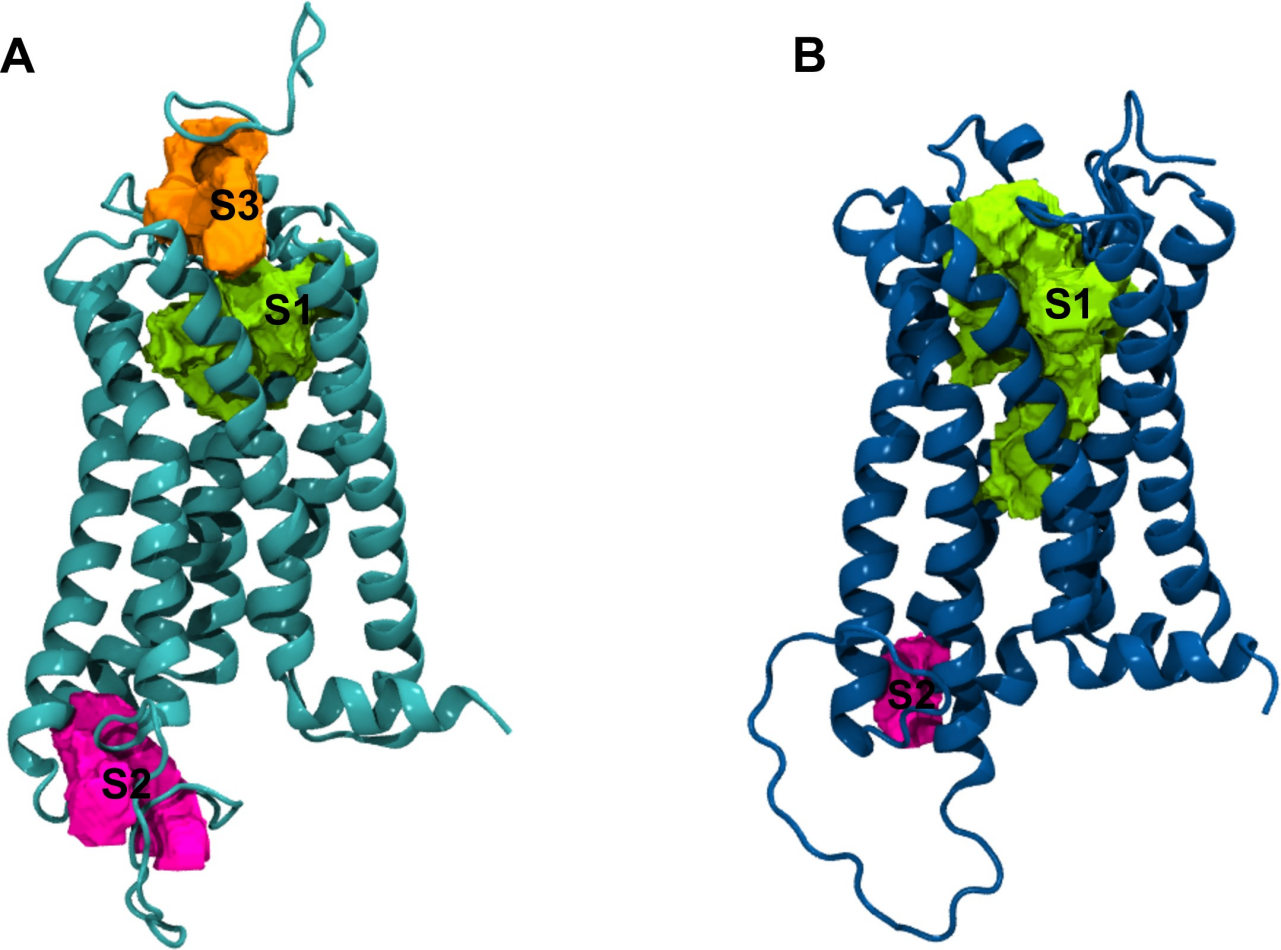
Binding sites identified in the CB_1_R. (A) Conformation of the CB_1_R bound to agonist. (B) Conformation of the CB_1_R bound to antagonist. Orthosteric site S1 (green) and potential allosteric binding sites S2 (magenta) and S3 (orange).

In order to identify possible allosteric binding sites in the CB_1_R generated models, the web servers Computed Atlas of Surface Topography of proteins (CASTp) and DoGSiteScorer were used. Two pockets were identified in the active and inactive receptor conformation and a third cavity was observed only in the active state receptor (**Fig 4**). **Table A in S1 File** shows the volume of the identified binding sites as calculated by the webserver DoGSiteScorer.

#### Site 1 (S1)

S1 was identified in both receptor conformations and corresponds to the orthosteric binding site. Reported co-crystallized orthosteric ligands occupy this cavity and in agreement with the crystallized CB_1_R structures, the volume of the ligand-binding pocket is approximately 50% larger in the inactive conformation than in the active conformation [9,10,53]

#### Site 2 (S2)

A second possible site was found between in the intracellular side of the receptor between TMH5, TMH6 and the ICL3. This region has been associated to coupling of G-protein during receptor activation and the structure of the ICL3 bound to G_αi_ protein has been determined [54–56]. The volume of S2 is larger in the active receptor conformation consistent with an increased surface area in the G-protein binding region during receptor activation.

#### Site 3 (S3)

A third binding pocket was found only in the active conformation of the receptor located in the transmembrane region between TMH3 and TMH7 and including part of the N-terminal region. This pocket sits above with the orthosteric site and has been previously reported as a putative allosteric pocket for other GPCRs [57]. The allosteric modulator ORG27569 was proposed to bind near this region where it could sterically block key electrostatic interactions involved in receptor activation [5,58].

From the three main pockets identified S1 corresponds to the orthosteric binding site and S2 can be related to the G-protein binding site. Therefore, only S3 was considered as a possible allosteric site for CBD and was selected for further docking studies. Furthermore, its location near the MPR can be linked to the reported effect of cysteine or polar residues in CBD allosteric modulation [59]. Although this relation was observed through functional assays and indirect mutagenesis studies, there is no clear evidence of a CBD allosteric binding site in this region nor of interactions between CBD and the disulfide bond.

### Molecular docking studies

In order to analyze possible binding modes and interactions of CBD in the selected pocket molecular docking studies were carried out. Although S3 was not identified in the inactive conformation, docking of CBD was also carried out for comparison. Because the co-crystallized ligands (AM11542 and AM6538) in both receptor conformations were analogues of either the partial agonist THC or the inverse antagonist rimonabant, docking of these ligands in the corresponding receptor conformation was first carried out. The resulting docking poses were in good agreement with the binding modes described for co-crystallized ligands with slight variation due to the modified motifs. The obtained protein-orthosteric ligand complexes were then used to perform docking of CBD. **Table B in S1 File** shows the lowest docking binding energy obtained for CBD in the CB_1_R in presence of the orthosteric ligands.

Results show that in both receptor conformations the lowest-energy complexes CBD binds to the extracellular portion, between the ECL1 and ECL3, near the MPR, a conserved region necessary for ligand binding [48,60] Nearby residues (< 6Å) include C107 and C98 forming the disulfide bind that has been linked to CBD and other allosteric modulators activity in the CB_1_R [31,60].

**Fig 5:**
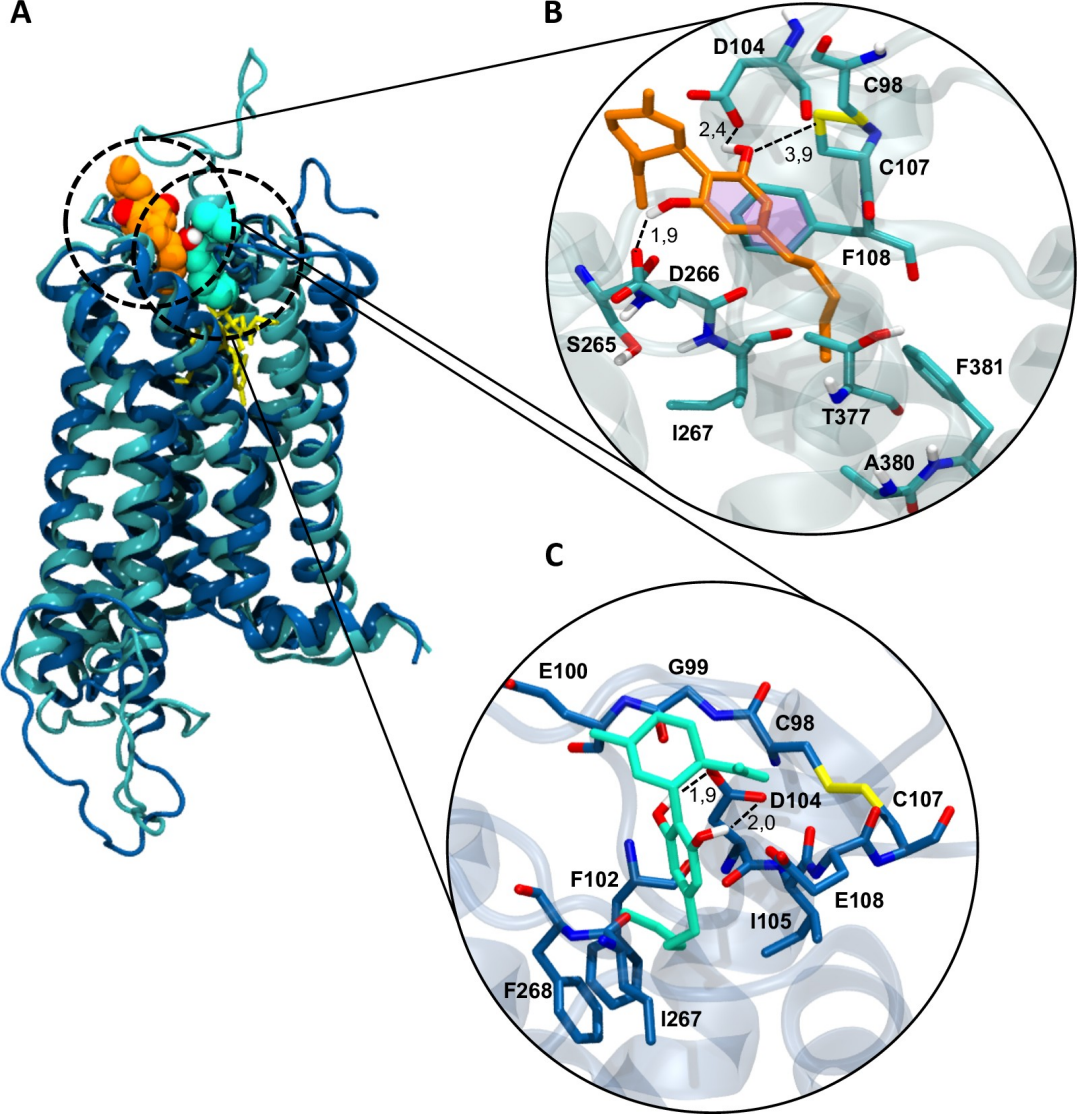
Docking conformations of CBD in the CB_1_R. (A) Superposition of the active (cyan) and inactive (blue) receptor conformation and vdW representation of CBD in orange and green respectively. The orthosteric ligands THC and rimonabant are shown in yellow sticks. (B) CBD (orange sticks) bound to the active receptor conformation. (C) CBD (green sticks) bound to the inactive receptor conformation. Nearby residues (<5 Å) are shown, polar interactions are indicated with dotted lines, aromatic interactions are indicated by pink surfaces and distances are expressed in Å.

In the agonist bound conformation, CBD binds in a solvent exposed cavity in the extracellular side near the N-terminal region and above the orthosteric site. Here CBD adopts a vertical disposition with the alkyl chain extending towards the transmembrane region. Nearby residues (<5 Å) are mostly non-polar but two aspartate residues, D104 and D266, lie close to CBD 1’,3’-hydroxyl groups and can establish ion-dipole interactions. The aromatic ring can form pi-stacking interactions with F108 and the alkyl chain interacts through van der Waals interactions with I91, A204 and F205. CBD 3’-hydroxil group and the C98-C107 disulfide bond is located 3,9 Å away from the C98-C107 disulfide bond and can form a dipole interaction. This dipolar interaction is in line with results reported by Lapraire *et al*., where mutation of cysteine residues to alanine abolished CBD modulating effects but replacement with serine restored the wild-type response. **Fig 5** summarizes the binding mode and interactions observed for CBD in the active and inactive receptor conformation.

On the other hand, in the antagonist-bound conformation folding of the N-terminal tail allows CBD to enter deeper into the receptor partially overlapping with the orthosteric site. The alkyl chain of CBD is turned towards residues F102, I105, I267 and F268 establishing hydrophobic contacts. Similar to the active conformation, CBD hydroxyl groups can form interactions with D104 and E106, but the disulfide bond is farther away (>6Å) making the probability and intensity of an interaction with these residues less likely.

If CBD can act as an allosteric modulator of THC effects, then its binding to the CB_1_R would be expected to occur in presence of an orthosteric ligand. The identification of the cavity S3 only in the active receptor structure suggests that allosteric ligand binding occurs or is favored in the active conformation stabilized by an orthosteric agonist. This is further supported by docking results that suggest CBD can interact with the C98-C107 disulfide bond in the active but not in the inactive conformation of the CB_1_R.

### Molecular dynamics (MD) simulation

To study the dynamic behavior of the obtained protein-ligand complexes MD simulations were carried out.

#### MD in the inactive conformation of the CB_1_R

The identified binding sites suggest CBD can bind in the active conformation of the CB_1_R, however, docking studies predict a lower binding energy in the inactive receptor conformation. For this reason, a short 25 ns simulation was run on the CBD-rimonabant-CB_1_R complex. The specific goals of this simulation were to: (1) observe if the interactions between the receptor, the orthosteric antagonist and CBD, as proposed by the docking studies persisted throughout the simulation and; (2) observe changes in the orthosteric binding site S1 and the possible formation of an allosteric site S3.

Analysis of the orthosteric ligand rimonabant shows that its position is preserved throughout the simulation time showing a similar initial and final conformation (RMSD 0,675 Å). Likewise, the interactions proposed by docking studies persist in the simulation and correspond well to those described in the antagonist-bound crystal structure of the receptor (see **Figure C in S1 File**). These are mainly hydrophobic interactions between the orthosteric ligand and residues from the ECL2, N-terminal and all TMs except TM4 [9].

**Fig 6:**
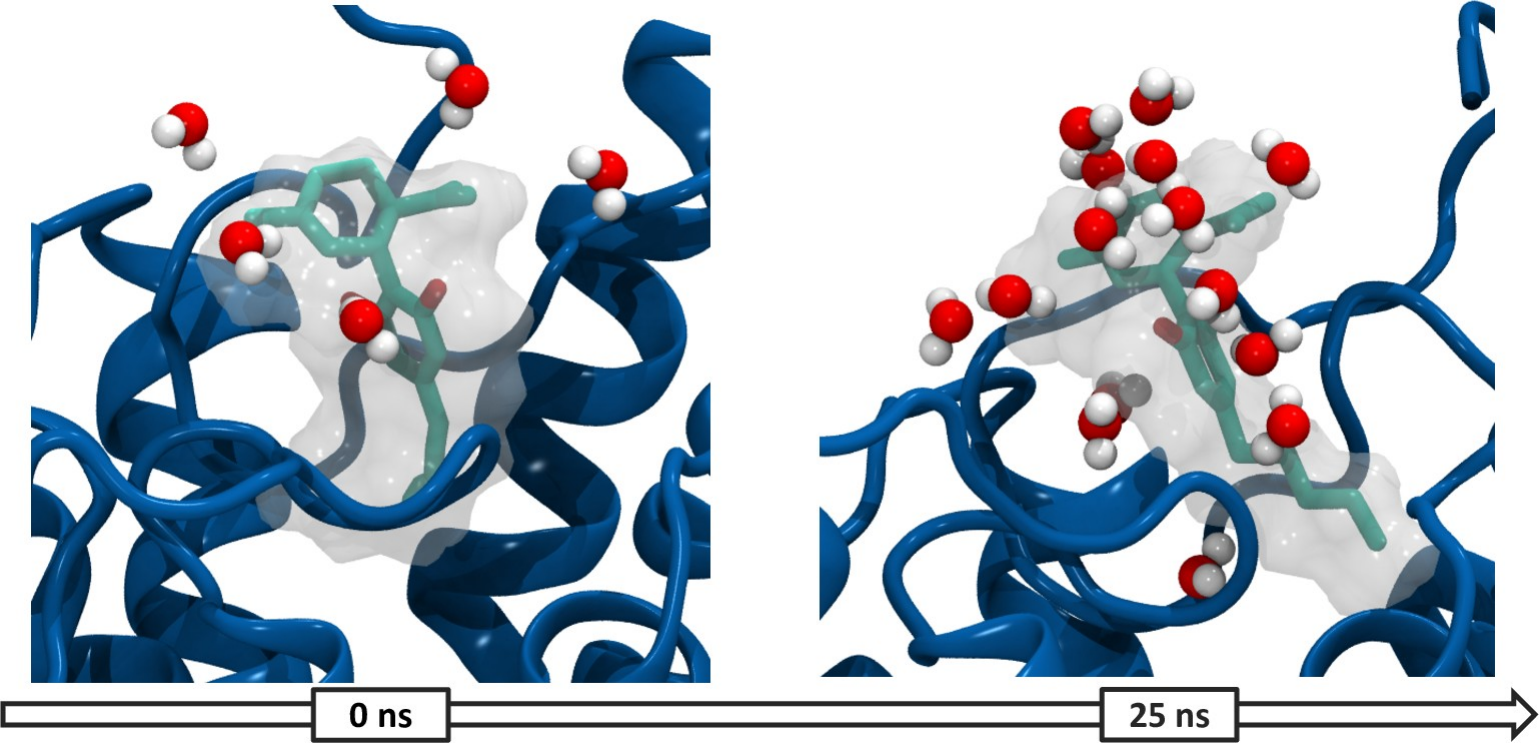
Solvent rearrangement around CBD in the antagonist bound CB_1_R. CBD (cyan) and water molecules within ≥3 Å in the inactive conformation of the CB_1_R at 0 and 25 ns of simulation.

Regarding the allosteric ligand, position and interactions are not retained in the simulation. Initial docking conformation shows CBD bound in the N-terminal region with its alkyl chain folded over the orthosteric site, however, throughout the simulation the alkyl chain is seen to extend and turn as CBD moves ~2,5Å towards the extracellular side. This movement can be explained by a solvation; simulation reveals that water molecules enter through the extracellular side and solvate CBD reorganizing the hydration shell in the ligands surface. At the same time, movement of water molecules from the bulk solvent into CBD binding site guide the rearrangement of its hydrophobic alkyl chain as it turns towards TMs 5 and 6 (**Fig 6**).

Therefore, the interactions between CBD and the receptor proposed in the static model are replaced by interactions with the solvent. Specifically, two hydrogen bonds between CBD and residues D104 and E10 are replaced for hydrogen bonds with water molecules. On the other hand, analysis of the binding sites shows that the orthosteric pocket S1 undergoes minimal changes and retains its total volume (see **Table A in S1 File**) and S3 is not formed during the simulation time.

These results suggest antagonist binding stabilized the CB_1_R in a rather rigid conformation in which binding of CBD seems to be unstable throughout time. Rearrangement of water molecules that solvate the allosteric ligand seems to represent an entropic cost associated to CBD binding that is not compensated by the interactions formed with the receptor. At the same time folding of the N-terminal tail over the orthosteric site prevents the formation of a pocket that can bind CBD in this region and blocks the access into the receptor. These findings are in line with the observed modulation effects of CBD over the agonist THC and further support that CBD allosteric binding occurs only in the active conformation and in presence of an orthosteric agonist.

#### MD in the active conformation of the CB_1_R

To analyze CBD binding in the active receptor conformation a second simulation of 25 ns was run on the CBD-CB_1_R-THC complex. The specific goals of this simulation were to: (1) observe if the interactions between the receptor, the orthosteric agonist and CBD as proposed by the docking studies persist throughout the dynamic simulation; (2) observe changes in the orthosteric S1 and allosteric S3 binding sites and; (3) observe the interaction between residues F200 and W356 that form the ‘twin-toggle switch’.

**Fig 7:**
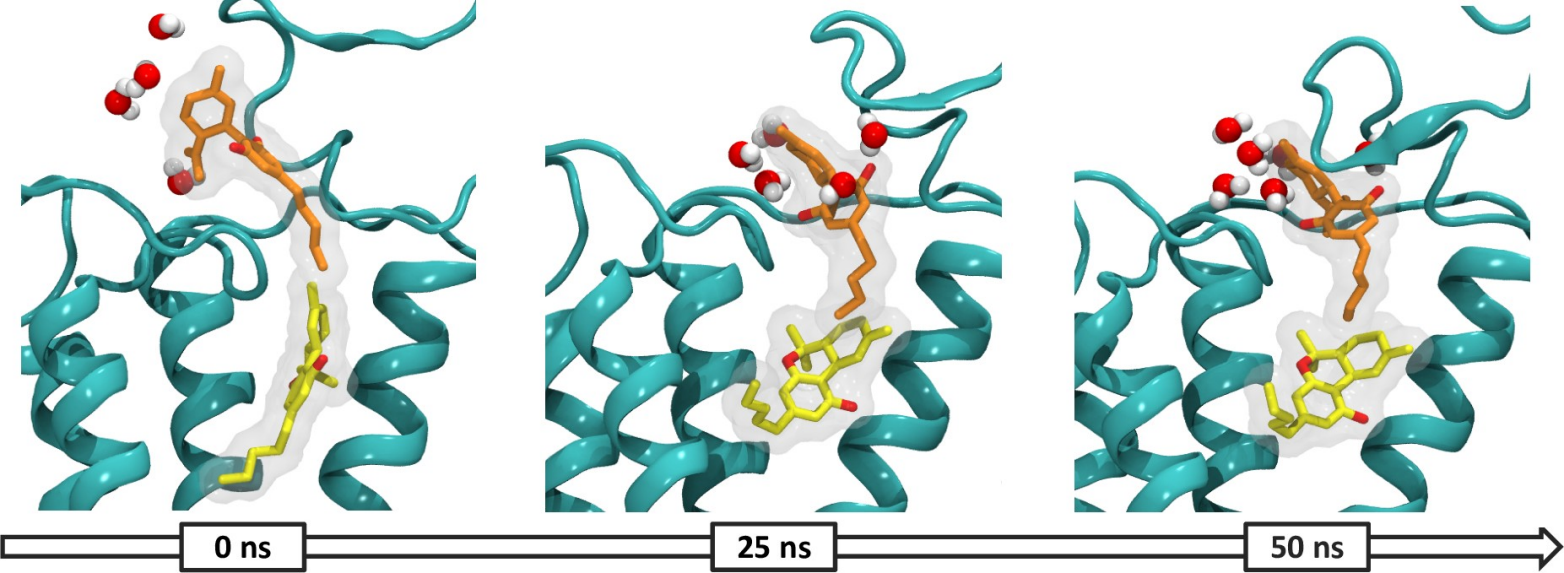
Solvent rearrangement around CBD in the agonist bound CB_1_R. CBD (orange), THC (yellow) and water molecules within ≥3 Å in the active conformation of the CB_1_R at 0, 25 and 50 ns of simulation.

More movement and changes were observed in the active conformation, so the simulation time was extended to 50 ns. In general terms, results show a coordinated motion that generates the opening of the cytoplasmic and extracellular sides and allows accommodation of the ligands in the binding site. Analysis of the ligands reveals a coordinated movement between CBD and THC that involves; solvation of the exposed terpenic ring of CBD by water molecules and folding of the MPR over site S3, which together promote partial entry of CBD into the orthosteric binding site and the accommodation of THC which adopts an L-shape conformation (**Fig 7**).

The N-terminal loop formed by the C98-C107 disulfide bond closes over CBD as it enters deeper into the binding site and in solvated. In this disposition CBD interacts mainly with the ECL2 and the N-terminal region and forms hydrogen bond with I267 and water molecules. An interaction network between the disulfide bond, CBD and water molecules that guides CBD entry into the binding site could explain the importance of polar residues in positions 98 and 107 for the modulating effects described by Laprairie *et al*.

THC maintains mainly hydrophobic and aromatic interactions; the tricyclic core forms aromatic interactions with P177, P189 and P268 while the pentyl side chain extends towards TM 3, 5 and 7. The L or C-shape conformation adopted during the simulation is consistent the conformation predicted for the endogenous ligands 2-Arachidonoylglycerol (2-AG) and Anandamide (AEA) [9].

**Fig 8:**
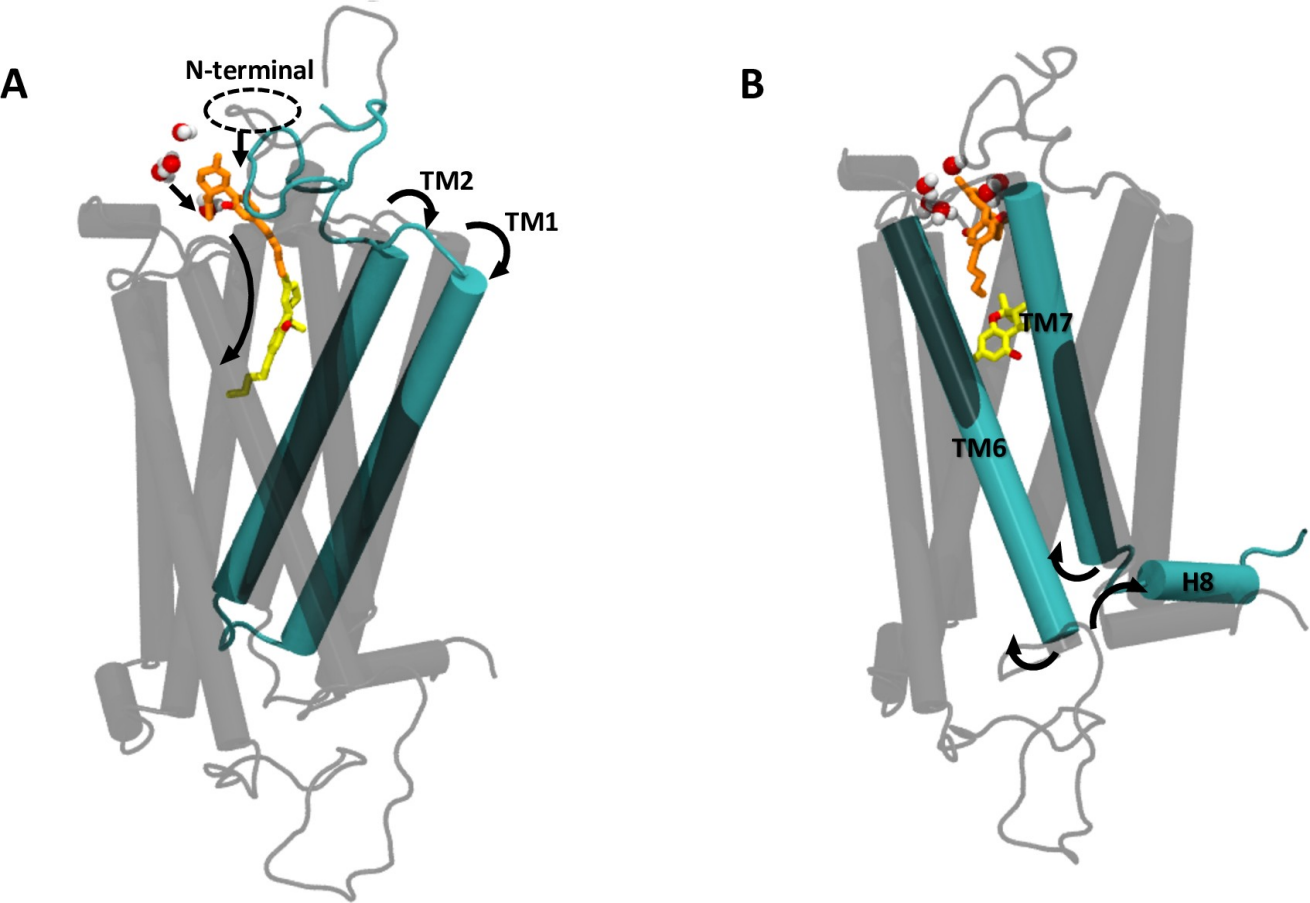
Coordinated movement observed in the active conformation of the CB_1_R during 50 ns of simulation. (A) Major movement in the extracellular and (B) intracellular side. CBD (orange), THC (yellow) and water molecules within ≥3 Å of CBD are shown.

The RMSD values for each helix showed that TM 1, 2, 6 and 7 were more dynamic (see **Figure D in S1 File**). Helixes 1 and 2 rotate outwards in the extracellular side while in the intracellular side helixes 6 and 7 move outwards. Rotation of TMs 1 and 2 and consequent opening in both sides of the receptor allows the expansion of the orthosteric binding site and the subsequent ligand accommodation. **Fig 8** summarizes the coordinated movement observed during the simulation.

**Fig 9:**
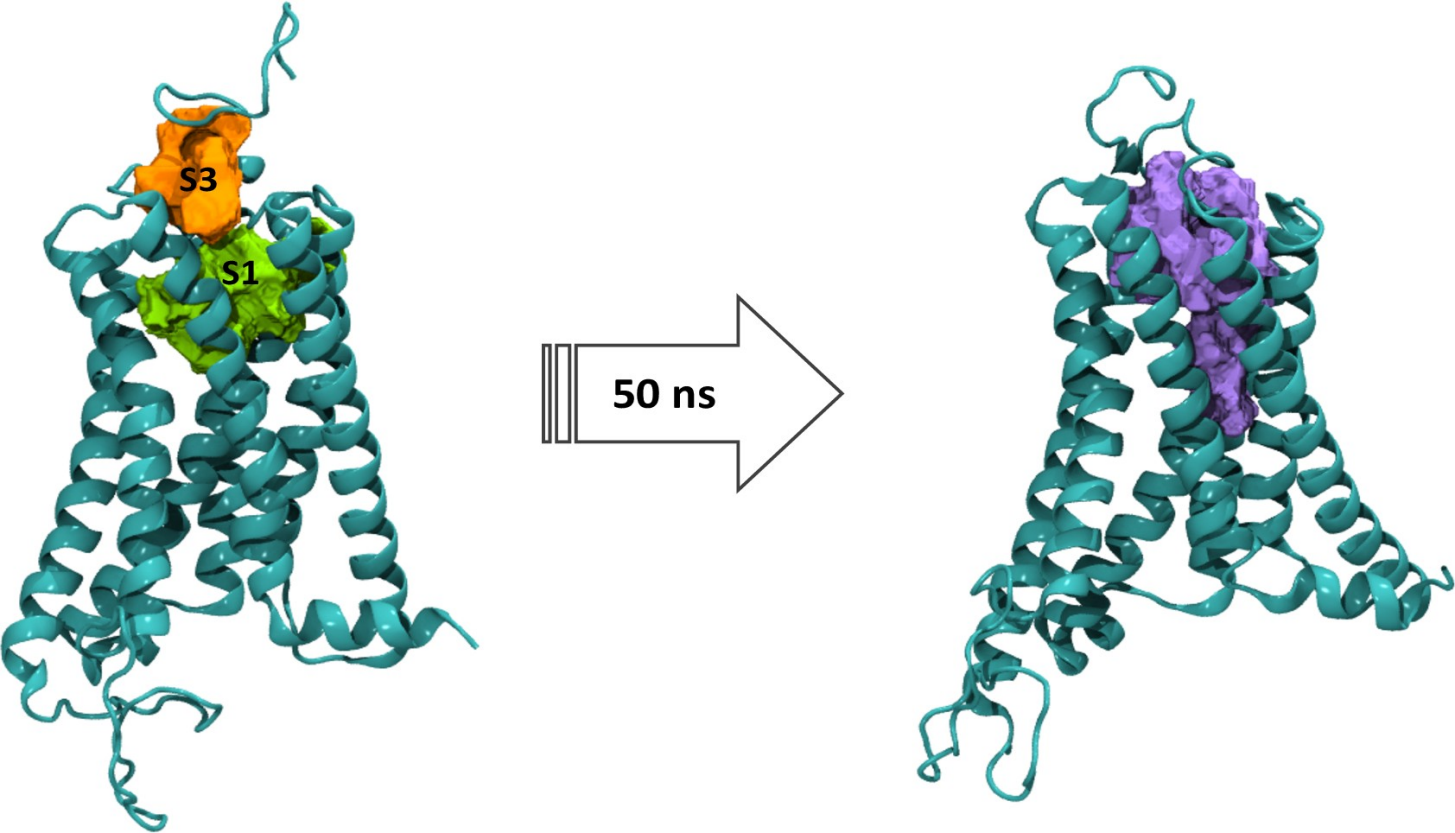
Changes in the binding sites S1and S3 in the active conformation of the CB_1_R after 50 ns of simulation. Orthosteric site S1 (green), allosteric site S3 (orange) and combined pocket (purple).

Analysis of the binding pockets in the active conformation state shows that S1 and S3 expand and combine into one larger pocket where CBD and THC can be found (**Fig 9**). The contribution of multiple binding sites in the allosteric mechanism of the cannabinoid PAM ZCZ011 has been described by Saleh *et al*. [61] and overlapping of different binding sites has also been proposed as a possible mechanism of receptor modulation.

**Fig 10:**
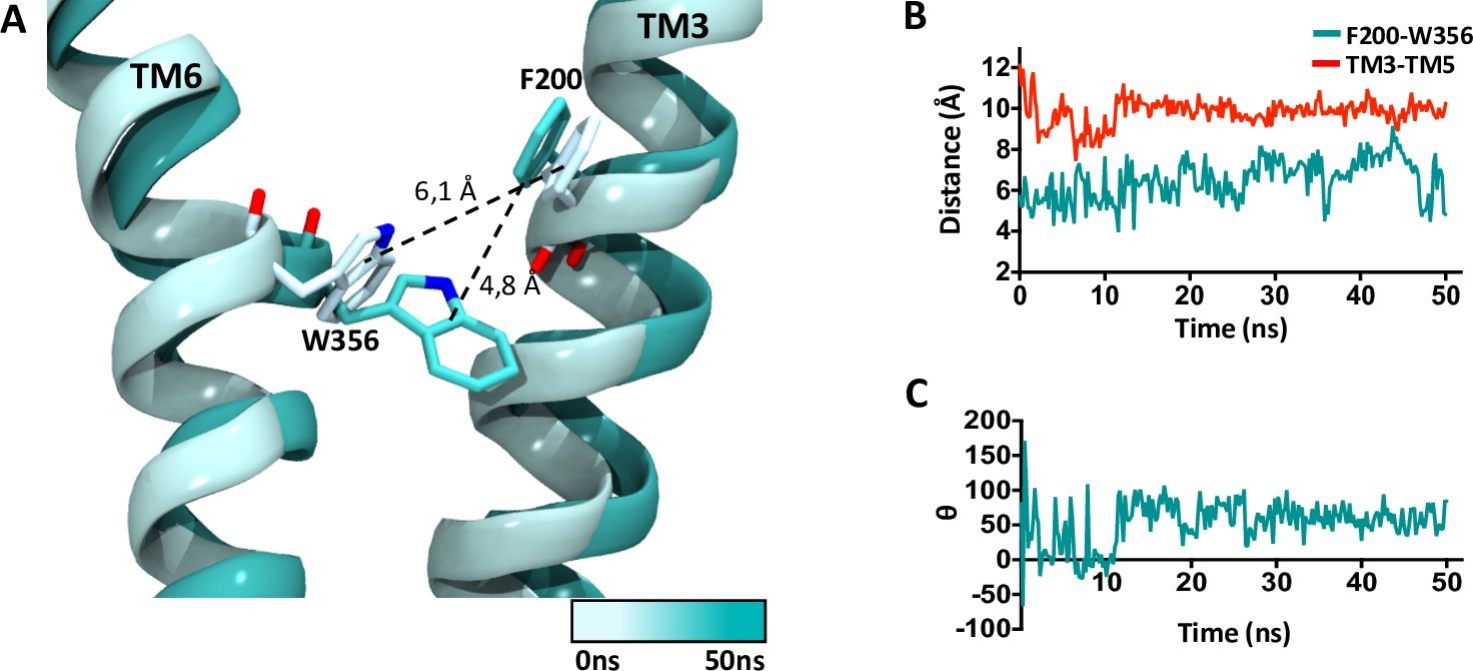
Twin-toggle switch interaction. (A) Interaction between F200 and W356 in the active conformation of the CB_1_R bound to THC and CBD. (B) Distance between aromatic centroids (cyan) and Cα (red) of residues F200 and W356. (C) Dihedral angle between F200 and W356 ring planes throughout 50 ns of simulation.

The aromatic interaction between F200 and W356 contributes to stabilization of the receptor in the inactive state but is disrupted in the active conformation [10]. This interaction has been described as a twin-toggle switch essential for receptor activation, for this reason, movement of these residues was also analyzed in the simulation. **Fig 10** shows the distance between TM 3 and 6, the aromatic centroids of F200 and W356 as well as the dihedral angle formed by the two ring planes throughout the simulation time.

Analysis of close by residues shows a network of polar threonine (T197, T391) and serine (S199, S203, S390) residues that seem to induce the reorientation of aromatic residues while the outward rotation of helix 5 allows TMs 3 and 6 to move closer together. In this way, at the end of the simulation the aromatic rings of F200 and W356 are separated by <5 Å forming a perpendicular angle which makes an aromatic edge-to-face interaction likely. Therefore, in agreement with the role of a twin-toggle switch and the NAM effect observed for CBD, our results suggest that binding of CBD in the active conformation of the CB_1_R promotes the formation of this aromatic interaction and could in this way prompt a transition towards and inactive receptor state.

**Fig 11:**
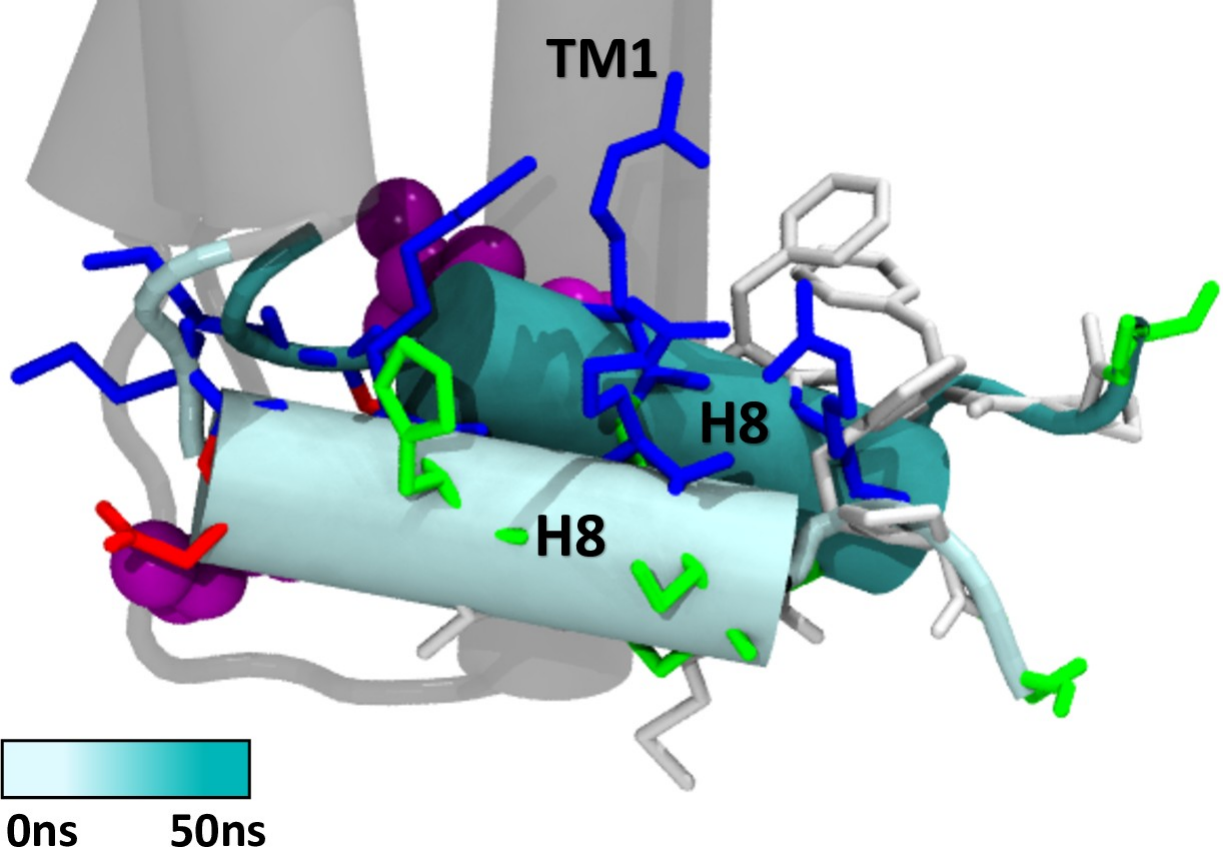
Movement of helix 8 in the active conformation of the CB_1_R throughout 50 ns of simulation. Residue type is indicated in color code: acidic residues (red), basic residues (blue), polar residues (green) and non-polar residues (white). L404 is shown in vdW representation (purple).

Another dynamic site is observed in helix 8 of the receptor (residues 403–413), an amphipathic helical domain within the C-terminal that has been identified as a conserved structural motif in class A GPCRs [62,63]. During the simulation helix 8 is seen to rotate orienting its hydrophobic face towards TM 1 (**Fig 11**). The movement of a leucine residue in this region is noteworthy. L404^7.60^ forms part of the highly conserved NPXXY(X)5,6 GPCR motif and has been described as essential for selective coupling to G-protein subtypes in the CB_1_R [64]. MD results show that at the beginning of the simulation L404 points towards the cytoplasmic region but at the end of the simulation, rotation of helix 8 buries this residue against TM 1 (**Fig 11**). Site-directed mutagenesis studies have shown that L404 regulates receptor internalization rate and is important to achieve maximal receptor activation in response to bicyclic cannabinoid agonists and inverse antagonists [64].

Changes in the orientation of helix 8 together with reduced exposure of residue L404 in the cytoplasmic region could explain the NAM effect of CBD observed by Laprairie *et al*. Although β-arrestin binding site and phosphorylation sites described for the cannabinoid receptor involve the distal C-terminus (a region not included in our receptor structures) flexibility seen in the immediately adjacent helix 8 could modulate exposure of phosphorylation sites and in this way alter β-arrestin recruitment and receptor internalization process. Consistent with this notion, high mobility in helix 8 is required in the low-affinity arrestin binding state of the rhodopsin receptor [65].

The dynamic behavior of helix 8 highlight the importance of having a complete CB1R structure that includes the distal C-terminal region. Although the receptor models used in this work lack the complete C-terminus they contribute relevant information that can help to guide future studies.

## Conclusion

In this work, allosteric modulation of CBD in the CB_1_R was studied based on the possibility of its direct receptor blockade and its effect on the conformational changes associated to the activation and inactivation of the receptor. The mechanism by which CBD exerts its cannabinoid effects remains in discussion and although different mechanisms, including allosteric modulation have been proposed, to date there is no evidence of the direct binding of CBD in an allosteric site of the CB_1_R.

In summary, results obtained in this work show that CBD is able to bind in an allosteric site of the CB_1_R and thereby promote conformational changes that can be associated to the transition towards an inactive or impaired signaling receptor state. An allosteric site was identified in the active receptor conformation that was able to bind CBD in a ligand-specific manner. We suggest dipolar interactions are essential for N-terminal movement and CBD stabilization in the binding site. MD simulations showed coordinated motions that promoted opening of the receptor extra and intracellular ends with a subsequent expansion of the binding pocket similar to an inactive state.

The role of the extracellular and intracellular regions suggests that incorporation of both segments is important for the study of allosteric mechanisms. Likewise, further studies in the distal C-terminal that can confirm the relation between mobility in helix 8 and receptor internalization will be useful to better understand the mechanism of CBD modulation in the CB_1_R. Other authors have suggested that CBD can antagonize the effects of THC through its interaction with other molecular targets, such as, the transient receptor potential vanilloid 1 (TRPV1), adenosine receptor (A_2A_) and serotonin receptors (5-HT_1A_). Proposed mechanisms have been extensively reviewed elsewhere [29].

Our studies allow us to rationalize the binding of CBD and offer a possible explanation to the effects of negative modulation observed in functional assays. Nevertheless, they do not exclude the possibility of an indirect modulation mediated by other molecular targets that, in conjunction with allosteric binding, contribute to the overall effects observed *in vivo*.

## Supporting information

S1 File (PDF)

S2 File (RTF)

S3 File (ZIP)
