# Supplementary material for: Cannabidiol binding and negative allosteric modulation at the cannabinoid type 1 receptor in the presence of delta-9-tetrahydrocannabinol: An In Silico study
Source: PLoS One. 2019 Jul 23;14(7):e0220025. doi: 10.1371/journal.pone.0220025 (PMC6650144; doi:10.1371/journal.pone.0220025)
Supplement: S1 File — (PDF) [file pone.0220025.s001.pdf]

Cannabidiol binding and negative allosteric modulation at the cannabinoid type 1 receptor in the presence of delta-9-tetrahydrocannabinol: an *in silico* study

Hery Chung[1], Angélica Fierro[2], C. David Pessoa-Mahana[1]*.

[1]Pharmacy Department, Faculty of Chemistry, Pontificia Universidad Católica de Chile, Santiago, Chile.
[2] Organic Chemistry Department, Faculty of Chemistry, Pontificia Universidad Católica de Chile, Santiago, Chile.

*Corresponding author
E-mail: cpessoa@uc.cl (CDP)

Supporting Information

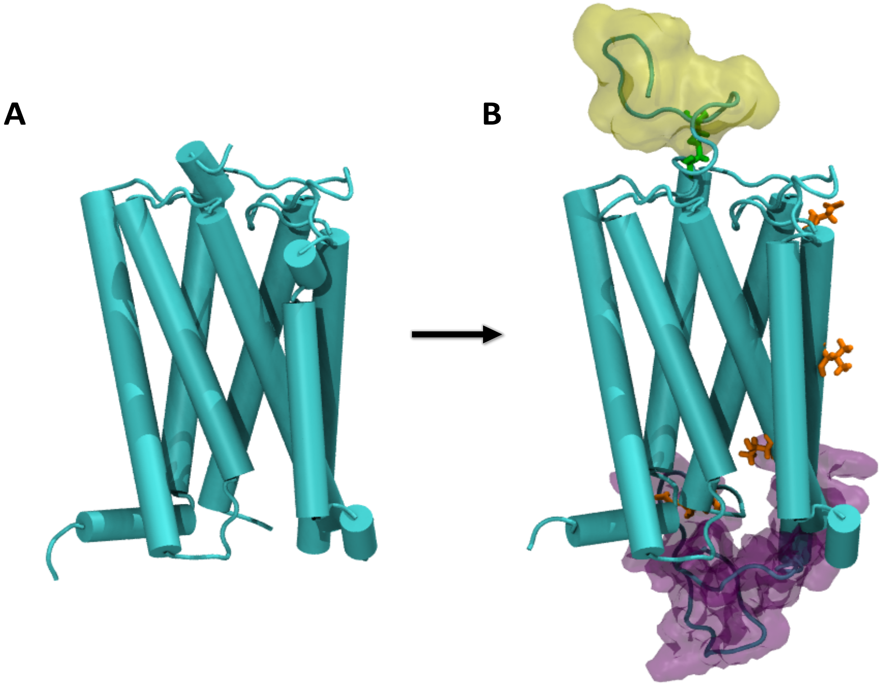

C

```
MKSILDGLAD TTFRTITTDL LYVGSNDIQY EDIKGDMASK LGYFPQKFPL  50
TSFRGSPFQE KMTAGDNPQL VPADQVNITE FYNKSLSSFK ENEENIQCGE 100
NFMDIECFMV LNPSQQLAIA VLSLTLGTFT VLENLLVLCV ILHSRSLRCR 150
PSYHFIGSLA VADLLGSVIF VYSFIDFHVF HRKDSRNVFL FKLGGVTASF 200
TASVGSLFLT AIDRYISIHR PLAYKRIVTR PKAVVAFCLM WTIAIVIAVL 250
PLLGWNCEKL QSVCSDIFPH IDETYLMFWI GVTSVLLLFI VYAYMYILWK 300
AHSHAVRMIQ RGTQKSIIIH TSEDGKVQVT RPDQARMDIR LAKTLVLILV 350
VLIICWGPLL AIMVYDVFGK MNKLIKTVFA FCSMLCLLNS TVNPIIYALR 400
SKDLRHAFRS MFPSCEGTAQ PLDNSMGDSD CLHKHANNAA SVHRAAESCI 450
KSTVKIAKVT MSVSTDTSAE AL                               472
```

Figure A. Three-dimensional structure of the human CB_1R in its active conformation. (A) Crystal structure of the CB_1R bound to agonist PDB: 5XRA. (B) Receptor structure generated by molecular modeling of missing segments. (C) Aminoacidic sequence of the human CB_1R. Modelled segments are indicated with colors; N-terminal region (yellow), ICL3 (purple), disulfide bridge (green) and mutated residues (orange).

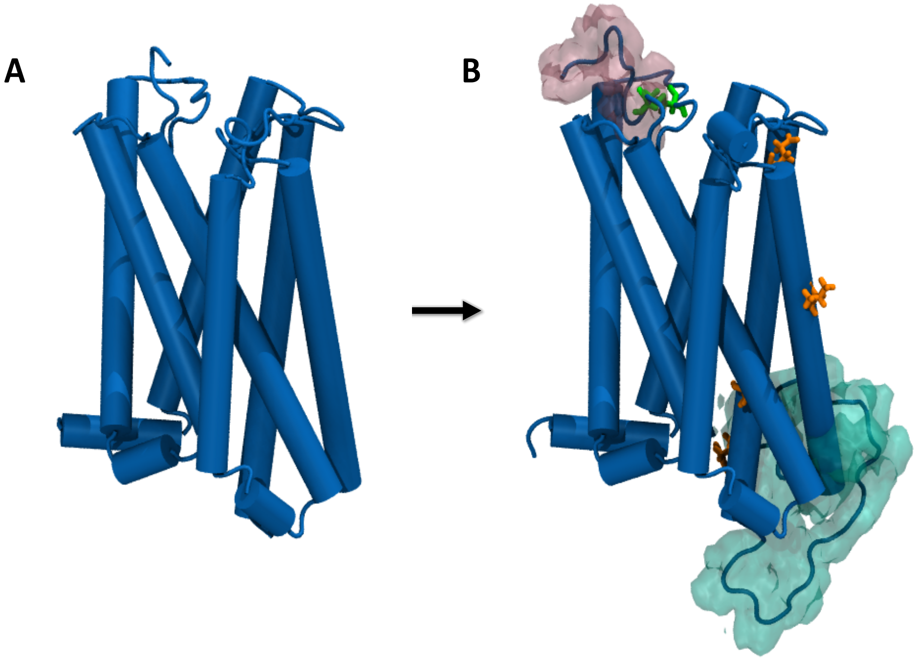

C

```
MKSILDGLAD TTFRTITTDL LYVGSNDIQY EDIKGDMASK LGYFPQKFPL  50
TSFRGSPFQE KMTAGDNPQL VPADQVNITE FYNKSLSSFK ENEENIQCGE 100
NFMDIECFMV LNPSQQLAIA VLSLTLGTFT VLENLLVLCV ILHSRSLRCR 150
PSYHFIGSLA VADLLGSVIF VYSFIDFHVF HRKDSRNVFL FKLGGVTASF 200
TASVGSLFLT AIDRYISIHR PLAYKRIVTR PKAVVAFCLM WTIAIVIAVL 250
PLLGWNCEKL QSVCSDIFPH IDETYLMFWI GVTSVLLLFI VYAYMYILWK 300
AHSHAVRMIQ RGTQKSIIIH TSEDGKVQVT RPDQARMDIR LAKTLVLILV 350
VLIICWGPLL AIMVYDVFGK MNKLIKTVFA FCSMLCLLNS TVNPIIYALR 400
SKDLRHAFRS MFPSCEGTAQ PLDNSMGDSD CLHKHANNAA SVHRAAESCI 450
KSTVKIAKVT MSVSTDTSAE AL                               472
```

Figure B. Three-dimensional structure of the human CB_1R in its inactive conformation. (A) Crystal structure of the CB_1R bound to antagonist PDB: 5TGZ. (B) Receptor structure generated by molecular modeling of missing segments. (C) Aminoacidic sequence of the human CB_1R. Modelled segments are indicated with colors; N-terminal region (magenta), ICL3 (cyan), disulfide bridge (green) and mutated residues (orange).

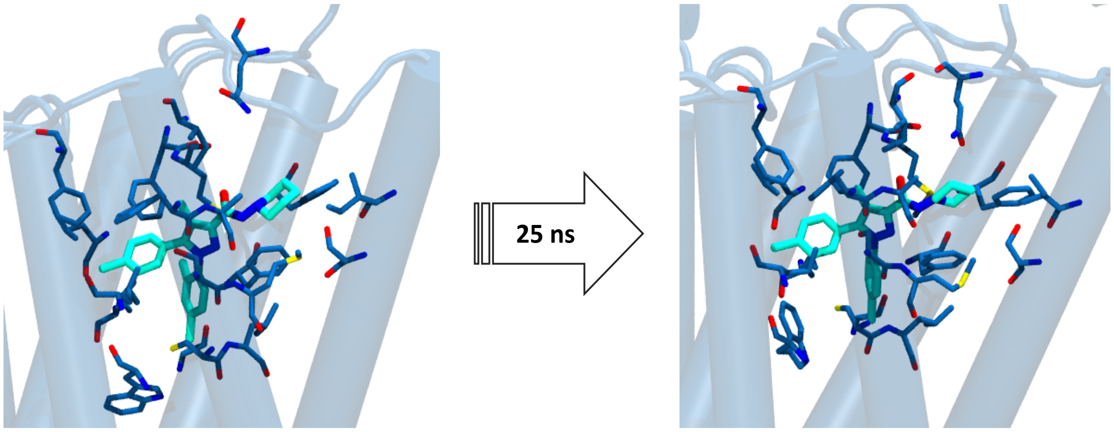

Figure C. Binding interactions of rimonabant in the inactive conformation of the CB_1R after 25 ns of simulation. The orthosteric ligand rimonabant and nearby residues (<5 Å) are shown in cyan and blue sticks respectively.

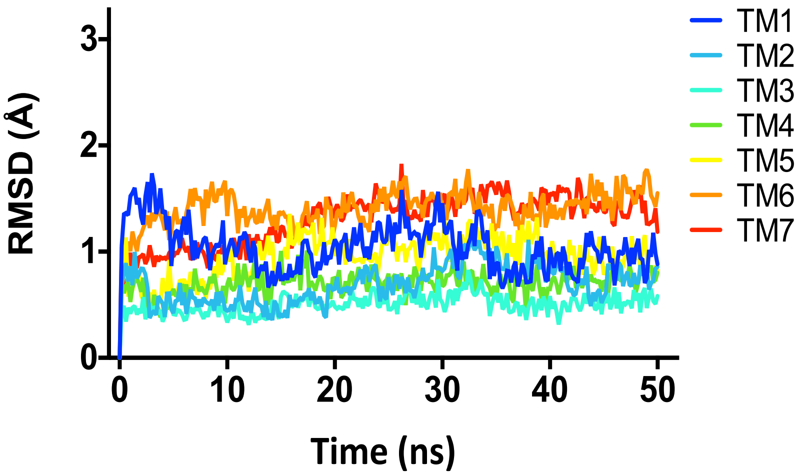

Figure D. Plot of RMSD values for each TM helix in the active conformation of the CB_1R.

Table A. Volume of the identified binding sites in the CB_1R bound to agonist and to antagonist.

Identified binding site	Volume (A^3)	
	**CB_1R-agonist**	**CB_1R-anatagonist**
S1	909,89	1508,47
S2	500,35	336,77
S3	618,94	Not identified

Table B. Lowest binding energies of the docking conformations obtained for CBD in the CB_1R.

**Ligand**	**CB_1R**	**Binding Energy** (kcal/mol)
CBD	Active conformation bound to THC	-6,09
CBD	Inactive conformation bound to rimonabant	-7,88
