# Supplementary material for: Cannabidiol binding and negative allosteric modulation at the cannabinoid type 1 receptor in the presence of delta-9-tetrahydrocannabinol: An In Silico study
Source: PLoS One. 2019 Jul 23;14(7):e0220025. doi: 10.1371/journal.pone.0220025 (PMC6650144; doi:10.1371/journal.pone.0220025)
Supplement: S2 File — (RTF) [file pone.0220025.s002.rtf]

Links to publicly available repositories are listed below.

1) Reported CB1 receptor crystal structures bound to agonist and antagonist were retrieved from Protein Data Bank (PDB). Accession codes ID: 5XRA and ID: 5TGZ.

https://www.rcsb.org/
https://www.rcsb.org/structure/5xra
https://www.rcsb.org/structure/5tgz


2) Human CB1 receptor sequence was retrieved from the UniProt Knowledgebase database (UniProtKB). Entry P21554.

https://www.uniprot.org/
https://www.uniprot.org/uniprot/P21554


3) Topology and parameter files for the ligands were generated using the web server SwissParam

http://www.swissparam.ch/


4)  Orientation of the receptor structures with respect to the membrane were obtained from the Orientations of Proteins and Membranes (OPM) database. Accession codes 3495 and 3243

https://opm.phar.umich.edu/
https://opm.phar.umich.edu/proteins/3495
https://opm.phar.umich.edu/proteins/3243
